# Non-infectious osteomyelitis of the mandible in a young woman: a case report

**DOI:** 10.1186/1752-1947-8-44

**Published:** 2014-02-12

**Authors:** Anne Q Rasmussen, Ulrik B Andersen, Niklas R Jørgensen, Peter Schwarz

**Affiliations:** 1 Research Center of Ageing and Osteoporosis, Department of Medicine M, Glostrup University Hospital, 2600 Glostrup, Denmark; 2 Research Center of Ageing and Osteoporosis, Department of Diagnostics, Glostrup University Hospital, 2600 Glostrup, Denmark; 3 Faculty of Health Sciences, University of Copenhagen, Copenhagen, Denmark; 4 Osteoporoseambulatoriet MAO og Forskningscenter for Aldring og Osteoporose FAO, Medicinsk afd. M, Glostrup Hospital (Nord), Ndr. Ringvej, 2600 Glostrup, Denmark

**Keywords:** Bisphosphonate, Bone, Non-infectious osteomyelitis, Zoledronic acid

## Abstract

**Introduction:**

We present the case of a patient with non-infectious osteomyelitis of the mandible, which is a recognized but unusual condition of unknown cause.

**Case presentation:**

A 14-year-old Caucasian girl presented with pain and edema in the left side of her jaw. A clinical examination led to a diagnosis of osteomyelitis and she was treated with antibiotics. Our patient continued antibiotic treatment for osteomyelitis and underwent decortication. Histology based on a biopsy showed new bone formation and chronic inflammation, and a diagnosis of sclerotic osteomyelitis was made. Over the next few years, she experienced pain on the left side of her jaw and increasing edema, and the size of the left side of her jaw bone increased. She was then sent to our Department of Medicine at the age of 16 years. Her symptoms included pain in the left side of her jaw that scored 4 on a visual analogue scale of 1 to 10. A diagnosis of bone disease was made based on bone scintigraphy and single photon emission computed tomography that showed hot spots in the affected left side of the jaw. Our patient was treated with a single dose of intravenous zoledronic acid (5mg) at age 17 years, which was repeated after 12 months. The bone pain was significantly reduced six months after treatment and had disappeared 24 months after treatment.

**Conclusion:**

We report an unusual localization of non-infectious osteomyelitis of the jaw in a young woman. Even though the presentation was in the jaw, her condition improved after intravenous bisphosphonate treatment, as evaluated by reduced clinical symptoms, bone turnover evaluation as assessed by biochemical bone markers, and reduced activity on bone scintigraphy.

## Introduction

Subacute and chronic symmetrical osteomyelitis was first described by Giedion *et al*. [1]. In this report, the disorder involved the metaphyses of long bones either simultaneously or successively. Later publications reported the involvement of additional bone sites and used the terms chronic multifocal osteomyelitis and chronic recurrent multifocal osteomyelitis (CRMO) [2,3]. The disease is rare, accounting for 2% to 5% of all osteomyelitis cases. It primarily affects children, with a female to male ratio of 5:1, with no racial predilection [4]. In a five-year follow-up study of 23 patients published in 2002, the median age of onset was 10 years with a reported range of 4 to 14 years [4]. Chun reported on a female patient who did not have any prior dental infection or skin manifestations associated with CRMO. That patient was treated with oral prednisone and alendronate, and naproxen (Naprosyn) as needed for pain [5]. However, after six and a half years of treatment, the patient had persistent facial deformity due to diffuse bilateral mandible enlargement. In this case and review by Chun, it was concluded that clinicians caring for children should be familiar with CRMO because it typically occurs during childhood and should be included in the differential diagnosis of patients presenting with signs and symptoms of unifocal osteomyelitis. Because other sites of bone involvement may be asymptomatic, a bone scan is recommended for patients with negative bacterial cultures from bone biopsies and for those presenting with mandibular inflammation.

We report on a case of unifocal non-infectious osteomyelitis in the mandible of a young woman.

## Case presentation

A 14-year-old Caucasian girl presented to her local dentist with pain and edema in the left side of her jaw, with a maximum mouth opening of 31mm. Histology and a clinical examination resulted in a diagnosis of osteomyelitis and she was treated with antibiotics. She then underwent surgical decortication at a hospital department of dental surgery. Histology based on a biopsy showed new bone formation and chronic inflammation, and she was diagnosed with sclerotic osteomyelitis. There was no clinical improvement after the decortication, and over the next few years she experienced pain in the left side of her jaw and increasing edema, and the size of the left side of her jaw bone increased.

Our patient was admitted to our Department of Medicine at the age of 16 years. Her symptoms included pain in the left side of her jaw that scored a 4 on a visual analogue scale of 1 to 10. There was a visible difference in the two halves of our patient’s face, with increased soft tissue and edema on her left side. She had menarche at 13 years of age and regular menses. There was no change of pain during her menstrual cycle. Our patient had normal plasma C-reactive protein (<10mg/L), plasma calcium ion (1.23mmol/L) and plasma parathyroid hormone (1–84) (29ng/L), but low plasma 25-hydroxyvitamin D (vitamin D; 23nmol/L; reference range >50nmol/L) and high plasma alkaline phosphatase (122U/L; reference range 35 to 105U/L). Her weight was 58.3kg, height 168.6cm and body mass index 20kg/m^2^. Her bone mineral density was measured and found to be normal (T-score of spine +0.1 and hip −0.3).

**Figure 1:**
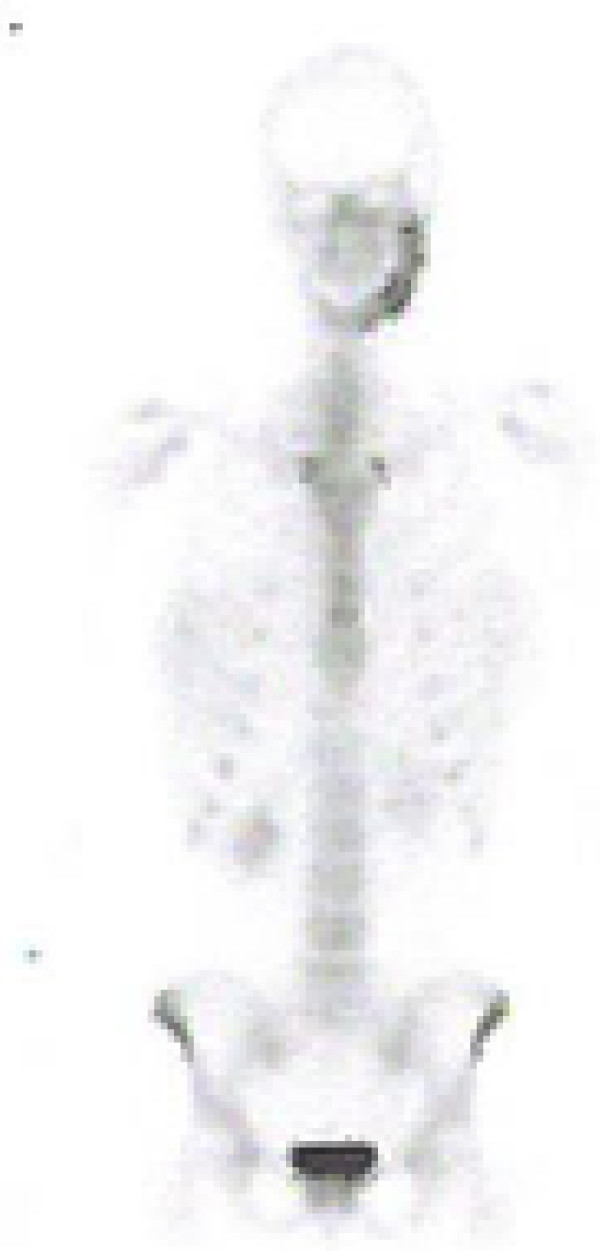
Bone scintigraphy of the patient at month 0.

**Figure 2:**
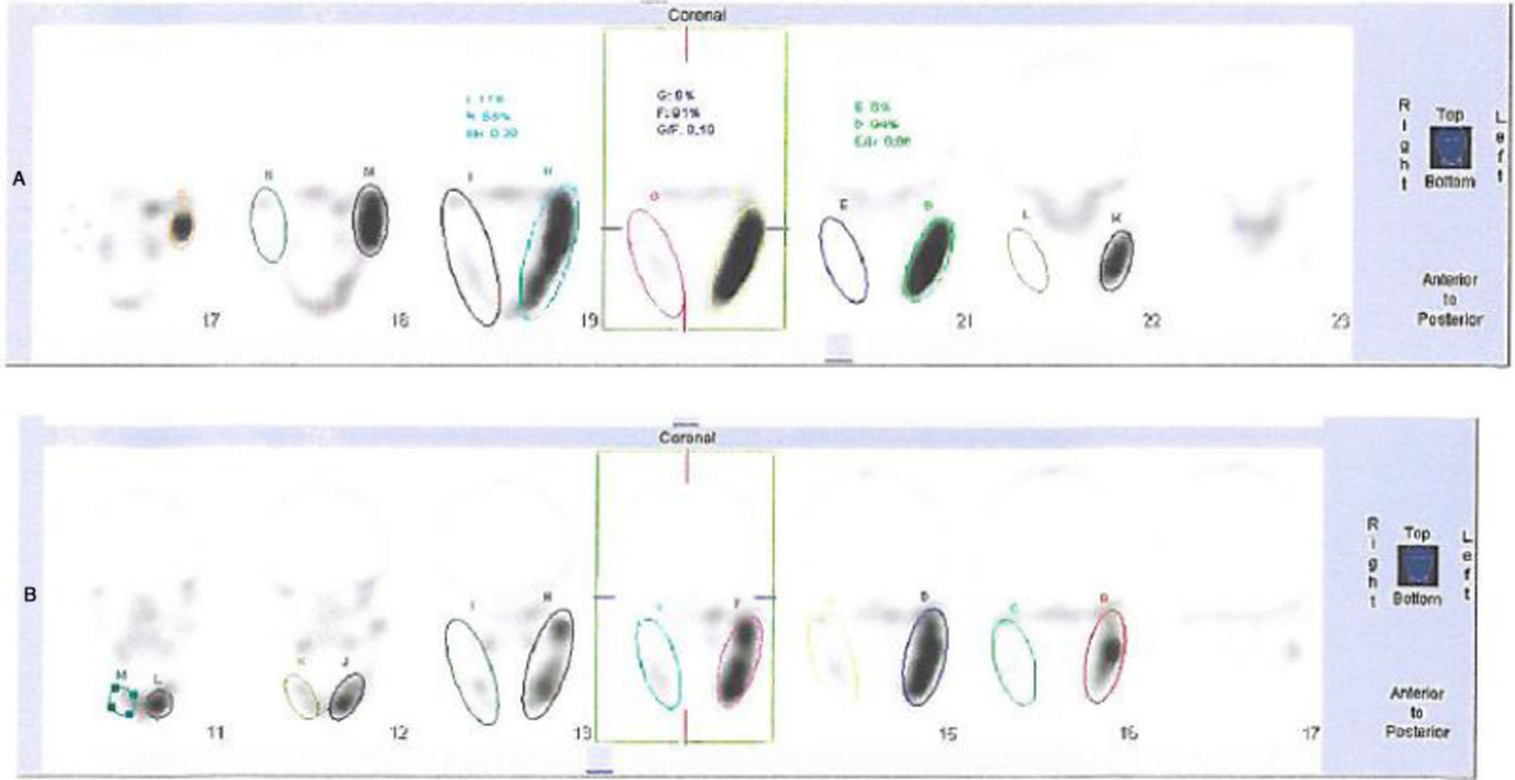
Single photon emission computed tomography of the jaw at month 0 before treatment, panel A (left-right ratio 1:16.4) and after 24 months of zoledronic acid treatment, panel B (left-right ratio 1:4.4).

A diagnosis of bone disease was made based on bone scintigraphy (Figure 1), single photon emission computed tomography (SPECT) (Figure 2) showing hot spots in the affected side of the jaw, and the above biochemistry. This led to a re-analysis of the bone biopsy by two specialists. The biopsy did not allow an assessment of any increased activity by osteoclasts and osteoblasts. Bone remodeling was disorganized, with persistence of woven bone and most likely diagnostic of non-infectious osteomyelitis.

Our patient was initially treated with vitamin D until the deficit had corrected. This had no effect on the disease. Thereafter, at age 17, she was treated with a single 5mg dose of intravenous zoledronic acid (Aclasta) that was repeated after 12 months. After 24 months of treatment, the left-side soft tissue and facial edema of our patient remained unchanged. However, her bone pain was significantly reduced six months after the first zoledronic acid treatment and had disappeared 24 months after treatment. The maximal opening of her mouth was unchanged after six months of treatment, but had increased from 31mm to 63mm after 24 months of treatment. Biochemistry at 24 months showed normalized plasma alkaline phosphatase (73U/L; reference range 35 to 105U/L) and unchanged C-reactive protein (<10mg/L; within the reference range). SPECT at 24 months of treatment showed less activity in the affected hot spot on the left side of her jaw (right-left ratio 1:16.4 at baseline and 1:4.4 after 24 months of treatment, Figure 2). Control markers of bone turnover were suppressed: plasma CTX, 0.04μg/L (reference range: 0.11 to 0.74μg/L) and plasma osteocalcin, 2.4μg/L (reference range: 10.4 to 45.6μg/L). Bone alkaline phosphatase, was within the normal range at 11.6μg/L (reference range: 4.7 to 27.0μg/L).

## Discussion

CRMO is a relapsing inflammatory disease affecting a variety of bones such as the mandible, spine, pelvis, sternum and scapula in addition to the originally described long bones [3]. The reported cases are all multifocal and only few adult cases has been reported [5].

We have presented a case of unifocal bone disease in the mandible. The histological report of woven bone was non-specific as were the abnormalities on isotope scan and SPECT. However, these findings could be caused by non-infectious osteomyelitis of the mandible, which is a recognized but unusual condition of unknown cause.

The biopsy in our case confirmed our diagnosis of osteomyelitis. Furthermore, osteomyelitis was suspected in our patient at age 14. She was treated by relevant antibiotics for several months but with no clinical response. Evaluations by SPECT, bone scintigraphy and biochemistry provided no definitive diagnosis. A diagnosis of non-infectious osteomyelitis was assumed but could not be confirmed. Our case has several similarities to the review and case presented by Chun, even though our patient’s disease had, throughout the years, only presented as a unifocal non-infectious osteomyelitis [5].

The differential diagnosis for our patient had included Paget’s disease of the bone (PDB) [6]. However, the age of our patient and the biopsy evaluation were against this diagnosis, despite one of three specialists suggesting a diagnosis of PDB. Early-onset familial PDB would be an outside possibility, but with no family history it seemed unlikely. Another possible explanation could have been fibrous dysplasia or other anomaly of bone structure.

We decided to treat our patient’s bone abnormality with bisphosphonate and chose zoledronic acid. Another option could have been pamidronate, a less potent bisphosphonate. Even though our patient was young, we chose the most potent bisphosphonate as it was assumed that she might only be treated once and, in cases of PDB, it has been observed that a single treatment with zoledronic acid can reduce bone activity for several years. Our case demonstrates that zoledronic acid rapidly decreases bone activity as evaluated by biochemistry and by bone SPECT. The clinical improvement in pain was significant at month six of treatment. The treatment also led to increased mouth opening, which more than doubled from 31mm to 63mm by month 24 of treatment, thereby significantly improving quality of life for our young female patient.

## Conclusion

We have described an unusual localization of non-infectious osteomyelitis of the jaw in a young woman. Even though the presentation was in her jaw, the bone condition improved after intravenous bisphosphonate treatment, as evaluated by reduced clinical symptoms, bone turnover evaluation assessed by biochemical bone markers, and reduced activity on bone scintigraphy.

## Consent

Written informed consent was obtained from the patient’s legal guardians for publication of this case report and any accompanying images. A copy of the written consent is available for review by the Editor-in-Chief of this journal.

## Abbreviations

CRMO: chronic recurrent multifocal osteomyelitis; PDB: Paget’s disease of the bone; SPECT: single photon emission computed tomography.

## Competing interests

The authors declare that they have no competing interests.

## Authors’ contributions

PS treated our patient and observed her during the follow-up period. UBA performed scintigraphy assessments. AQR and NRJ analyzed biomarkers and participated together with PS in analyzing the data. PS was the major contributor in writing the manuscript. All authors read and approved the final manuscript.
